# One-year post-discharge health-related quality of life in digestive and oncology patients: a three-group comparison by nutritional status and care

**DOI:** 10.1007/s11136-025-04139-y

**Published:** 2025-12-26

**Authors:** Josune Martín, Nere Larrea, Yolanda García, Inmaculada Bolinaga, Amaia Perales, Cristina Sarasqueta, José M. Quintana

**Affiliations:** 1 Department of Neuroscience, University of the Basque Country UPV/EHU, 48940 Leioa, Spain; 2 Research Unit, Galdakao-Usansolo Hospital, Barrio Labeaga s/n, 48960 Galdakao, Bizkaia Spain; 3 Department of Endocrinology, Galdakao-Usansolo Hospital, Barrio Labeaga s/n, 48960 Galdakao, Bizkaia Spain; 4 Biosistemak Institute for Health Systems Research, Barakaldo, Bizkaia Spain; 5 Research Network on Chronicity, Primary Care and Prevention and Health Promotion (RICAPPS), 48960 Galdakao, Bizkaia Spain; 6 BioBizkaia Health Institute, Barakaldo, Bizkaia Spain; 7 Biodonostia Health Institute, Gipuzkoa, Spain; 8 Donostia Hospital, Gipuzkoa, Spain

**Keywords:** Health-related quality of life, Hospitalization, Malnutrition, Nutritional care, One year follow-up

## Abstract

**Purpose:**

We evaluated changes in health-related quality of life (HRQoL) twelve months after admission among three groups of patients with digestive and oncological pathologies by nutritional status and nutritional intervention. We also sought to identify factors influencing those changes, taking into account the patients’ baseline status.

**Methods:**

This pragmatic effectiveness trial study comprised 619 hospitalized patients who completed HRQoL instruments on admission and twelve months later. Of these, 365 were classed as non-malnourished and 254 as malnourished (of whom 59 underwent nutritional intervention). Sociodemographic and clinical data were gathered. Patients completed the Short-Form 36; the EuroQol generic health-related quality of life questionnaire and the Barthel Index. Descriptive, univariate and multivariate linear regression models were applied to identify factors associated with changes in HRQoL.

**Results:**

Participants had a mean age of 63.5 years; 61.6% were male. Malnourished patients showed significant improvement in mental, social and physical components at the change. In the multivariate analysis, several variables affected the HRQoL of hospitalized patients one year after admission, such as level of malnutrition, pathology on admission, gender, age, Charlson Comorbidity Index, number of drugs prescribed, length of stay or surgical intervention. The nutritional intervention provided during the days of admission was not found to be associated with better HRQoL, except for role physical and body pain.

**Conclusion:**

Identifying the factors influencing changes in HRQoL amongst patients one year after their admission could help determine how important it is to improve these factors, when possible, for example through nutritional intervention prior to admission for surgery.

**Supplementary Information:**

The online version contains supplementary material available at 10.1007/s11136-025-04139-y.

## Abstract (plain language summary)

Many people who are hospitalized with digestive or cancer-related illnesses also struggle with poor nutrition, which can affect their overall well-being. This study looked at how patients’ quality of life changed one year after being admitted to the hospital, especially in relation to their nutritional status and whether they received any nutritional support during their stay.

The main goal was to understand if being malnourished or receiving nutritional help made a difference in how patients felt physically, mentally, and socially over time. The study followed 619 patients and found that those who were malnourished showed noticeable improvements in several areas of life quality after a year. However, the nutritional support given during their hospital stay only had a small effect, helping mainly with physical roles and pain.

The results suggest that many factors—like age, gender, type of illness, and how many medications a person takes—can influence recovery and well-being. This means that improving nutrition before surgery or hospital admission might be more helpful than focusing only on care during the hospital stay, although it is important to take into account the limited effect of in-hospital intervention and the importance of pre-admission/preventive nutrition.

**Supplementary Information:**

The online version contains supplementary material available at 10.1007/s11136-025-04139-y.

## Introduction

HRQoL or Health-Related Quality of Life is a subjective evaluation of an individual’s well-being, which addresses not only physical aspects, but also level of independence, social relationships and psychological state [1]. Patient HRQoL is a very important gauge of outcomes; patients’ physical and emotional state while combatting illness can have a major effect on their capacity to perform everyday functions and impacts their interpersonal relationships and their ability to work [2].

HRQoL is significantly impacted by chronic and debilitating diseases (such as cancer, pancreatitis, and Crohn’s disease) and their associated complications, including malnutrition [3]. This is important, given that malnutrition affects at least 30% of patients with malignancies [4]; in fact, malnutrition in cancer patients is associated with poorer quality of life, increased morbidity and reduced survival rates [5]. Systematic reviews and meta-analyses investigate the effect of different nutritional interventions on mental health and QoL outcomes [6–9]. Nutritional support, when used appropriately, has a number of clinical benefits, including improved intake and nutritional status, functional recovery, fewer complications and reduced mortality [9–13]. Nutrition plays a crucial role in both preventing and alleviating the side effects associated with cancer treatment, making it a fundamental pillar in supporting their HRQoL. It has been shown that diet improvements significantly contribute to enhancing patients’ quality of life by positively influencing their emotional well-being, social interactions, and overall enjoyment of life [14]. This is largely because better nutritional status is strongly linked to a more favorable perception of health and general well-being. In Spain, the prevalence of hospital malnutrition ranges from 24% to 62% [15, 16]. While hospital undernutrition can be treated, it is often underdiagnosed and inappropriately addressed [17]; moreover, HRQoL and functional independence can be affected among people who have had a hospital admission, so that quality of life is of critical importance in chronic diseases and its measurement should be integrated as one main intervention target [18]. For this reason, we consider that it is of interest to analyze HRQoL among patients after hospitalization and the factors that influence their perception of health, particularly in the long term. We hypothesized that some variables, clinicals and sociodemographicals, can negatively impact the HRQoL one year at admission of patients and that being old, being female, or being hospitalized for surgical intervention are related to worse HRQoL one year after admission. The objectives of our study were to assess changes twelve months after admission in HRQoL among malnourished patients (whether or not they were specifically treated for that condition) and non-malnourished patients, hospitalized with diverse digestive and oncological diseases, and to identify factors that influence those changes, taking into account patient status at baseline, given that identifying the factors influencing changes in HRQoL amongst hospitalized patients one year after their admission could help determine how important it is to improve these factors. We point out that it is important to highlight the novelty of the paper, such as the study’s attempt to identify baseline factors that potentially influence changes in quality of life adds valuable insight to the existing literature.

## Methods

### Participants

We conducted a pragmatic effectiveness trial study of patients with oncological and digestive pathologies in three public hospitals in Spain: the Galdakao-Usansolo University Hospital, which serves a population of around 300,000 urban and semi-urban inhabitants; the Basurto University Hospital, which serves the urban population of Bilbao, (around 350,000 inhabitants); and the Donostia University Hospital which caters to the city of San Sebastián-Donostia and surrounding areas, with an overall urban and semi-urban population of around 400,000. The three hospitals have similar human and technological resources, and the population they cater to has similar sociodemographic and clinical characteristics. The project was evaluated and approved by the research committees of all participating centers and approved by the accredited Clinical Research Ethics Committee for the Basque Country (CEIC) (Approval Number: PI2018181) and registered in ClinicalTrials.gov as ID NCT04188990.

#### Inclusion criteria

Patients selected for the study were aged over 18 and had signed the informed consent form. All subjects met the inclusion criteria and none of the exclusion criteria. They were recruited consecutively in the first 48 h following admission to the Digestive, Oncology or Surgery Services of each center. We sought to recruit patients with a variety of pathologies: A - Digestive pathologies: acute pancreatitis, ulcerative colitis, and Crohn’s disease; B - Oncological pathologies: esophageal, gastric, pancreatic, colon and rectum cancers; and C) ulcerative colitis and Crohn’s disease admitted with surgical intervention.

#### Exclusion criteria

The following were excluded from the study: patients who had surgery for colon or rectum cancers detected via screening; patients with serious organic or psychopathological problems or in a terminal situation; patients admitted to short-term or critical care units; pregnant women; patients with neurosensory issues; patients who did not understand Spanish or were unable to complete the questionnaires used in the study; and patients who failed to sign the informed consent form.

### Procedure

During the first 72 h following admission, all patients admitted to the three centers who met the selection criteria were evaluated individually using the Malnutrition Universal Screening Tool (MUST) and the Global Leadership Initiative on Malnutrition (GLIM) questionnaires [19, 20]. Testing was performed in the Endocrinology and Nutrition Services by two trained nutritionists (one assigned to Galdakao and Basurto hospitals, and one to Donostia hospital). Three groups of patients were identified for the study, based on GLIM criteria: non-malnourished patients; malnourished patients who received nutritional intervention; and malnourished patients who received standard care. At that time, nutritional intervention was only administered at Galdakao-Usansolo Hospital, so all patients at this hospital with moderate or severe malnutrition (measured using GLIM criteria) were considered candidates for nutritional intervention; all other patients received standard care. In all patients, outcome measurements and clinical data were assessed at admission and 12 months after hospitalization.

Nutritional Intervention was performed between 24 and 48 h after the nutritional assessment (which was conducted 24–72 h after admission). The intervention was performed on an individual basis by the Endocrinology and Nutrition Service at Hospital Galdakao-Usansolo, by a specialist doctor and a nurse or nutritionist. A follow-up was performed every 24–48 h. On discharge, patients either received advice on adopting an oral diet or were prescribed oral supplements, enteral nutrition or parenteral nutrition to take at home, with subsequent monitoring by their GP, oncologist, surgeon or endocrinologist, depending on the specific pathology (see supplementary eFig. 1, Online Resource).

### Instruments and data collection

#### Sociodemographic, clinical and nutritional data

We retrieved the following information from the clinical history at admission, during admission, and at the 12-month follow-up: *sociodemographic data*: age, gender; *clinical data*: main pathology (esophageal cancer, gastric cancer, pancreatic cancer, colorectal cancer, colitis, Crohn’s, pancreatitis), severity of pathology (TNM if tumor), surgery intervention, hospital of admission, comorbidities based on the Charlson Comorbidity Index, treatments prescribed, length of hospital stay, infectious complications during admission, readmission at 90 days, mortality; *nutritional data*: handgrip strength as measured by handgrip dynamometer (Jamar Hydraulic Hand Dynamometer), calf diameter, weight, and Body Mass Index (BMI).

#### Patient-reported measures

Patients also completed Spanish-language versions of the following questionnaires:

- *Barthel Index*. Assessment of Performance in Basic Activities of Daily Living: Barthel Index. The Barthel Index [21, 22] is a scale used for assessing performance in basic activities of daily living. It considers 10 basic functions and has a score of 0–100. A higher score is associated with a greater likelihood of being able to live at home with a degree of independence following discharge from hospital. Scores were categorized using four cutoff points: 75–100 as reference (no/mild disability), 50–74 (moderate disability), 25–49 (severe disability), and 0–24 (completely dependent).
- *EQ-5D*. The self-report version of the *EuroQol generic health-related quality of life questionnaire* [23, 24] comes in two parts: the EQ-5D- 5 L descriptive system (Scale I) and the EQ Visual Analogue scale (VAS) (Scale II). The descriptive system consists of five dimensions. In each one, the patient chooses one of five answers, indicating varying degrees of severity. The EQ VAS maps the respondent’s self-rated health on a 20-cm vertical, visual analogue scale. The scale ranges from 0 (worst imaginable state of health) to 100 (best imaginable state of health).
- *SF-36 Health Questionnaire v2*. This is a generic instrument for assessing HRQoL that detects both positive and negative health states, as well as exploring physical and mental health. The 36 questions cover eight dimensions of health status: physical function, physical role, body pain, general health, vitality, social function, role emotional and mental health. Scores can range from 0 to 100, with a high score indicating a better health status. Patients are asked to rate their health as compared to one year ago. The SF-36 has been translated into Spanish and validated in populations in Spain [25].

#### Statistical analyses

We performed a descriptive analysis of the entire sample and a univariate analysis to identify possible variables related to the degree of malnutrition-intervention and between responders, non-responders and those who had died at one year, using percentages and frequencies for categorical data, means and standard deviations for continuous variables. We also calculated the median and interquartile range for non-normally distributed variables. We assessed differences in the level of malnutrition/intervention and between the different hospitals using the Chi-square test for categorical variables and the ANOVA or Kruskal-Wallis non-parametric test for continuous variables.

For the multivariate analysis, we used multilevel multivariable linear models, taking the hospital effect into account. We calculated the estimated (β) and standard deviation (sd) for changes in the three patient-reported questionnaires. We determined the predictive capacity of the models using the R^2^ determination coefficient. No data imputation has been performed, since the complete questionnaires have been used. Only patients who responded at baseline and follow-up were analyzed. All effects were considered statistically significant at *p* < 0.05. R statistical software, version 4.1. was used to create the graphics of the supplementary material. All statistical analyses were performed using SAS Software, version 9.4 (SAS Institute, Inc., Carey, NC, USA), and R statistical software, version 4.1.

## Results

### Description of the patient population

The study sample comprised 2064 participants with digestive and oncological pathologies, all recruited between June 2020 and December 2021. Of these, 1051 met the selection criteria and completed the basal questionnaires and 619 completed the 12-month follow-up (365 non-malnourished patients, 59 moderately or severely malnourished patients with nutritional intervention, and 195 moderately or severely malnourished patients with standard care) (eFig. 2, Online Resource). A total of 182 (17.32%) patients died before the one-year follow-up. Those who died were older patients (average 69 years) with a higher proportion of patients with colorectal (39.4%), gastric (21.7%) and pancreatic (27.8%) cancer, lower weight (66.7 ± 14.2), BMI (24.2 ± 4.7), handgrip strength (24.0 ± 10.2) and calf diameter (33.9 ± 3.9) at admission, and worse scores in functional and HRQoL parameters. Non-responders were younger (56.2 ± 16.9), included a higher proportion of men (55.2%) and patients with pancreatitis diagnoses (34.7%) and had fewer prescribed treatments.

The mean age of the patients responding on follow-up was 63.5 (SD = 16.3) and 61.6% were male. In terms of clinical variables, there were no significant differences in gender, days of admission or infectious complications between patients who completed the questionnaires at follow-up (*n* = 619, 23.78%), patients who did not respond (*n* = 250, 58.90%) and patients who died (*n* = 182, 17.32%). Among the other variables, there were statistically significant differences between these three groups; with the exception of esophageal cancer and pancreatitis, more malnourished patients with standard care died (e.g., 56.41% of gastric cancer patients who died were malnourished with standard care). As regards HRQoL, patients who responded after one year had had a statistically significant better baseline quality of life and functional status than those who died and those who did not respond at follow-up, except in physical functioning of SF-36, where there were no significant difference between responders and non-responders (eTable 1, Online Resource). The median for initiation of nutritional intervention was the second day (interquartile range: 1–3), with a median duration of three days (interquartile range: 1–6).

### Univariate analysis of the sociodemographic, clinical, nutritional and quality of life data

Among non-malnourished patients, there was a higher proportion of surgical interventions (56.2%); malnourished patients who received nutritional intervention had a higher 90-day readmission rate (25.4%) and malnourished patients with standard care had longer stays (7 days vs. 6 days). In nutritional parameters, both groups of malnourished patients —those with nutritional intervention and those with standard care— had worse baseline scores (Table 1). In terms of HRQoL results, malnourished patients showed a significant improvement in mental, social and physical components of their quality of life at the change (Table 2). With regard to the Barthel Index, the graphs show that there are patients with moderate or severe malnutrition and without nutritional intervention who have low scores at baseline and improve at follow-up. On the other hand, there are other non-malnourished patients and malnourished patients with nutritional intervention and better baseline Barthel scores who score worse at follow-up; ultimately, all patients ended up with similar outcomes, although we observe no statistically significant differences between them (eFig. 3, Online Resource).

**Table 1:** Univariate analysis of the malnutrition-intervention groups: not malnourished patients, patients malnourished with nutritional intervention and with standard care

Variables	Total (N = 619)	Level of malnutrition-intervention		
		Not malnourished^1^ (N = 365)	Malnourished with nutritional intervention^2^ (N = 59)	Malnourished with standard care^3^ (N = 195)
*Sociodemographic and clinical data*				
Age*	63.5 ± 16.3	63.0 ± 15.5	64.5 ± 16.6	64.1 ± 17.5^†^
Gender (Male)	381 (61.6)	228 (62.5)	41 (69.5)	112 (57.4)
Main pathology		2,3	1	1
Esophageal cancer	12 (2.0)	3 (0.8)	1 (1.7)	8 (4.1)
Stage I-II	5 (41.7)	2 (66.7)	0 (0.0)	3 (37.5)
Stage III	4 (33.3)	0 (0.0)	0 (0.0)	4 (50.0)
Stage IV	3 (25.0)	1 (33.3)	1 (100.0)	1 (12.5)
Gastric cancer	56 (9.2)	26 (7.2)	10 (16.9)	20 (10.4)
Stage I-II	29 (51.8)	16 (61.6)	2 (20.0)	11 (55.0)
Stage III	24 (42.9)	9 (34.6)	8 (80.0)	7 (35.0)
Stage IV	3 (5.3)	1 (3.9)	0 (0.0)	2 (10.0)
Pancreatic cancer	27 (4.4)	9 (2.5)	7 (11.9)	11 (5.7)
Stage I-II	10 (37.0)	4 (44.4)	2 (28.6)	4 (36.4)
Stage III	10 (37.0)	2 (22.2)	4 (57.1)	4 (36.4)
Stage IV	7 (26.0)	3 (33.3)	1 (14.3)	3 (27.2)
Colorectal cancer	229 (37.0)	126 (34.5)	22 (37.3)	81 (41.5)
Stage I-II	113 (49.8)	70 (56.5)	10 (45.5)	33 (40.7)
Stage III	77 (33.9)	32 (25.8)	9 (40.9)	36 (44.4)
Stage IV	37 (16.3)	22 (17.7)	3 (13.6)	12 (14.8)
Colitis	53 (8.7)	24 (6.7)	7 (11.9)	22 (11.4)
Crohn’s	69 (11.3)	38 (10.6)	5 (8.5)	26 (13.5)
Pancreatitis	173 (28.3)	139 (38.6)	7 (11.9)	27 (14.0)
Surgery intervention	305 (49.3)	205 (56.2)^2,3^	19 (32.2)^1^	81 (41.5)^1^
Charlson Index^¥^	2.0 (0.0–3.0)	2.0 (0.0–3.0)	2.0 (1.0–3.0)	2.0 (1.0–3.0) ^**†**^
Number of drugs^¥^	4.0 (2.0–7.0)	4.0 (2.0–7.0)	3.0 (1.0–6.0)	4.0 (2.0–7.0) ^**†**^
Readmission at 90 days	74 (12.0)	35 (9.6)^2^	15 (25.4)^1,3^	24 (12.3)^2^
Days of admission^¥^	6.0 (4.0–8.0)	6.0 (4.0–8.0)^3^	6.0 (4.0–9.0)	7.0 (5.0–9.0)^1**†**^
Infectious complications	18 (2.9)	11 (3.0)	2 (3.4)	5 (2.6)
*Nutritional variables*				
Handgrip strength at admission*	27.4 ± 11.6	28.3 ± 12.2	27.7 ± 10.5	25.7 ± 10.7^**†**^
Calf at admission*	35.6 ± 3.7	36.7 ± 3.7^2.3^	34.3 ± 3.2^1^	34.1 ± 3.2^1**†**^
Weight at admission*	71.9 ± 16.0	77.2 ± 16.0^2.3^	65.2 ± 12.4^1^	63.9 ± 12.5^1**†**^
BMI at admission*	25.8 ± 5.	27.7 ± 5.2^2,3^	22.9 ± 3.4^1^	23.0 ± 3.9^1**†**^

^1^ : Patients who were not malnourished at the time of admission; ^2^ : Patients who were malnourished at admission and received a nutritional intervention during that admission; ^3^ : Patients who were malnourished at admission and received the standard care during that admission. *Mean ± standard deviation. ^¥^Median (IQR (interquartile range)). ^†^Kruskal-Wallis test. Only values with superscript are significant

**Table 2:** Descriptive analysis of not malnourished patients, patients malnourished with nutritional intervention and with standard care, by health-related quality of life variables.

Variables	Total (*N* = 619)	Level of malnutrition-intervention		
		Not malnourished^1^ (*N* = 365)	Malnourished with nutritional intervention^2^ (*N* = 59)	Malnourished with standard care^3^ (*N* = 195)
*Health-related quality of life questionnaires*				
Barthel				
Baseline*	95.5 ± 8.7	96.1 ± 7.8	96.0 ± 7.5	94.3 ± 10.3^†^
Follow-up*	94.4 ± 10.0	94.6 ± 9.9	94.6 ± 10.8	94.1 ± 9.9^†^
Change^¥^	0.0 (− 5.0 to 0.0)	0.0 (− 5.0 to 0.0)	0.0 (0.0–0.0)	0.0 (0.0–2.5) ^†^
EQ-5D				
Baseline*	0.8 ± 0.2	0.8 ± 0.2	0.8 ± 0.2	0.8 ± 0.2^†^
Follow-up*	0.9 ± 0.2	0.9 ± 0.2	0.9 ± 0.2	0.9 ± 0.2^†^
Change^¥^	0.0 (− 0.1 to 0.1)	0.0 (− 0.1 to 0.1)	0.0 (0.0–0.1)	0.0 (− 0.0 to 0.1) ^†^
SF36-mental health				
Baseline*	62.2 ± 17.8	64.3 ± 17.4^3^	59.5 ± 18.8	59.0 ± 17.7^1†^
Follow-up*	64.9 ± 16.8	64.8 ± 17.3	64.1 ± 15.6	65.3 ± 16.3^†^
Change^¥^	0.0 (− 8.0 to 12.0)	0.0 (− 8.0 to 8.0)^3^	4.0 (− 8.0 to 12.0)	4.0 (− 4.0 to 16.0)^1†^
SF36-Physical Functioning				
Baseline*	70.4 ± 28.2	72.2 ± 28.1^3^	73.5 ± 25.1	66.2 ± 28.8^1†^
Follow-up*	71.2 ± 28.7	71.3 ± 29.3	73.2 ± 25.5	70.3 ± 28.4^†^
Change^¥^	0.0 (− 10.0 to 10.0)	0.0 (− 10.0 to 5.0)	0.0 (− 15.0 to 12.5)	0.0 (− 10.0 to 15.0) ^†^
SF36-social functioning				
Baseline*	68.2 ± 31.0	72.7 ± 28.3^3^	64.2 ± 32.8	61.1 ± 33.8^1†^
Follow-up*	74.8 ± 27.8	73.9 ± 29.1	76.1 ± 25.4	75.9 ± 25.8^†^
Change^¥^	0.0 (− 12.5 to 25.0)	0.0 (− 12.5 to 25.0)^3^	6.3 (− 12.5 to 25.0)	12.5 (0.0–37.5)^1†^
SF36-physical role				
Baseline*	63.1 ± 33.8	68.3 ± 32.3^2.3^	55.5 ± 34.5^1^	56.0 ± 34.8^1†^
Follow-up*	68.1 ± 33.0	68.5 ± 33.4	72.8 ± 27.5	65.9 ± 33.6^†^
Change^¥^	0.0 (− 12.5 to 25.0)	0.0 (− 18.8 to 18.8)^2,3^	18.8 (− 6.3 to 37.5)^1^	6.3 (− 12.5 to 31.3)^1†^
SF36-emotional role				
Baseline*	82.6 ± 24.5	83.9 ± 23.5	79.8 ± 27.0	81.3 ± 25.3^†^
Follow-up*	79.4 ± 26.8	79.5 ± 26.9	81.6 ± 24.6	78.4 ± 27.2^†^
Change^¥^	0.0 (− 16.7 to 8.3)	0.0 (− 16.7 to 0.0)	0.0 (− 16.7 to 16.7)	0.0 (− 16.7 to 8.3) ^†^
SF36-vitality				
Baseline*	52.9 ± 20.6	55.8 ± 20.4^2.3^	49.7 ± 19.3^1^	48.5 ± 20.7^1†^
Follow-up*	55.7 ± 19.6	55.8 ± 20.5	55.4 ± 16.9	55.7 ± 18.7^†^
Change^¥^	0.0 (− 16.7 to 8.3)	0.0 (− 16.7 to 0.0)	0.0 (− 16.7 to 16.7)	0.0 (− 16.7 to 8.3) ^†^
SF36-body pain				
Baseline*	58.8 ± 33.3	60.0 ± 33.3	57.8 ± 34.6	56.9 ± 33.1^†^
Follow-up*	67.9 ± 29.5	66.2 ± 29.9	70.0 ± 28.5	70.4 ± 28.9^†^
Change^¥^	0.0 (− 12.5–32.5)	0.0 (− 12.5 to 25.0)	0.0 (− 12.5 to 45.0)	10.0 (− 10.0 to 35.0) ^†^
SF36-general health				
Baseline*	53.1 ± 19.6	54.3 ± 20.1	51.7 ± 19.4	51.3 ± 18.7^†^
Follow-up*	52.6 ± 21.8	52.9 ± 22.7	53.0 ± 20.7	51.7 ± 20.5^†^
Change^¥^	0.0 (− 15.0 to 10.0)	0.0 (− 15.0 to 10.0)	0.0 (− 10.0 to 15.0)	0.0 (− 15.0 to 15.0) ^†^

^1^ : Patients who were not malnourished at the time of admission; ^2^ : Patients who were malnourished at admission and received a nutritional intervention during that admission; ^3^ : Patients who were malnourished at admission and received the standard care during that admission. *Mean ± standard deviation. ^¥^Median (IQR (interquartile range)). ^†^Kruskal-Wallis test. Only values with superscript are significant

With regard to the domains of the SF-36, malnourished patients who received nutritional intervention scored similarly at follow-up to the other groups, including non-malnourished patients, and even better in some areas, for example in vitality or mental health than the “other cancer” patient group, although not to a statistically significant extent (eFig. 4, Online Resource).

### Multivariate analysis for HRQoL variables

We found that the two malnourished groups showed greater improvement one year after the intervention than the non-malnourished group in the social functioning area of the SF-36 (*p* = 0.03); this improvement was greatest in the group that received the nutritional intervention. In addition, malnourished patients with nutritional intervention showed greater improvement in the role physical and bodily pain domains (*p* = 0.001 and *p* = 0.04, respectively). Notably, the decrease in women’s scores was greater than in men’s in all the questionnaires assessed and the greater the number of drugs prescribed, the greater the decrease. Likewise, the greater the number of comorbidities, as reflected by the Charlson Comorbidity index, the greater the losses in all the areas studied in the SF-36 and in the EQ-5D. Older age was related to losses on the Barthel Index and SF36 - Physical Function. Finally, patients with inflammatory bowel diseases and pancreatitis had greater losses in the mental health area of the SF-36, and greater length of stay during admission negatively impacted EQ-5D scores (Table 3).

**Table 3:** Multivariable multilevel models for functional status and health-related quality of life variables of patients one year after hospitalization

Functional status and health-related quality of life outcomes										
	Barthel	EQ-5D	Physical Function	Role Physical	Body Pain	Vitality	Social Functioning	Role Emotional	Mental Health	General Health
	β (sd)	β (sd)	β (sd)	β (sd)	β (sd)	β (sd)	β (sd)	β (sd)	β (sd)	β (sd)
Intercept	61.54 (5.61) **	0.73 (0.03)**	75.05 (5.46) **	72.51 (6.29)**	64.37 (4.19)**	38.83 (2.99)*	64.94 (4.45) **	56.95 (4.80)**	50.69 (3.81) **	31.24 (3.10)**
Baseline domain	0.43 (0.052) **	0.28 (0.03)**	0.43 (0.03) **	0.36 (0.04)**	0.25 (0.04)**	0.43 (0.04)**	0.28 (0.03) **	0.38 (0.04)**	0.40 (0.04) **	0.56 (0.04)**
Study groups										
No malnutrition	Ref.	Ref.	Ref.	Ref.	Ref.	Ref.	Ref.	Ref.	Ref.	Ref.
Malnourished with nutritional intervention	1.01 (1.304) ^ns^	0.05 (0.02)^ns^	4.52 (3.32) ^ns^	14.97 (4.48)**	8.57 (4.20)*	4.04 (2.61) ^ns^	8.40 (3.94)*	6.68 (3.83) ^ns^	0.79 (2.26) ^ns^	2.43 (2.76) ^ns^
Malnourished with standard care	0.72 (0.843) ^ns^	0.02 (0.01) ^ns^	2.28 (2.0) ^ns^	2.29 (2.74) ^ns^	5.02 (2.55) ^ns^	3.33 (1.61)*	5.19 (2.38)*	0.85 (2.33) ^ns^	1.73 (1.40) ^ns^	0.64 (1.68) ^ns^
Admission pathologies										
Other cancers	–	–	–	–	–	–	–	–	Ref.	–
Colorectal cancer	–	–	–	–	–	–	–	–	0.80 (1.93) ^ns^	–
Colitis, Crohn’s, pancreatitis	–	–	–				–		− 5.93 (2.30)*	
Gender (Female)	− 3.19 (0.78) **	− 0.04 (0.01)*	− 6.23 (1.91) **	8.85 (2.05)**	7.50 (2.35)*	4.74 1.48*	− 6.28 (2.18)*	6.86 (2.16)*	− 4.47 (1.29)**	5.23 (1.54)*
Age	− 0.09 (0.03)*	–	− 0.38 (0.06) **	0.27 (0.08)*	–	–	–	–	–	–
Charlson comorbidity index	–	− 0.01 (0.003)*	− 1.24 (0.49) **	2.20 (0.66)*	1.93 (0.60)*	1.00 (0.38)*	− 1.45 (0.55)*	1.67 (0.55)*	− 1.31 (0.43)*	1.29 (0.40)*
Number of drugs prescribed	− 0.45 (0.122)*	− 0.01 (0.002) **	− 1.45 (0.29) **	0.90 (0.40)*	1.57 (0.34)**	0.79 (0.22)**	− 1.46 (0.31) **	0.97 (0.32)*	− 0.56 (0.19)*	0.99 (0.23)**
Length of stay (days)	–	− 0.002 (0.001)*	–	–	–	–	–	–	–	–
Surgical intervention	–	–	–	–	–		–	–	− 4.32 (1.74)*	–
**R** ^**2**^	0.24	0.28	0.27	0.46	0.18	0.30	0.21	0.20	0.28	0.36

*p < 0.05; **p < 0.0001; ns: non statistical significant

## Discussion

This study sought to evaluate the degree and correlation of HRQoL and functional status for three groups of patients (non-malnourished patients, malnourished patients with nutritional intervention and malnourished patients without nutritional intervention) one year after hospital admission. In terms of clinical and sociodemographic variables, being female, being malnourished and having a larger number of prescribed drugs were associated with poorer quality of life after admission in all HRQoL questionnaires. Being older, having a longer hospital stay, having a surgical intervention or having colitis, Crohn’s disease or pancreatitis were also associated with a poorer quality of life but not in all HRQoL questionnaires. According to the results of the study, the hypothesis was partially accepted because not only domains of HRQoL was affected.

This study shows that 41% of hospitalized patients are malnourished (moderate or severe malnutrition) on admission (Stage 1 or 2 of GLIM criteria). This result is higher than the results of a recent study in Spain, which showed that 29.7% of hospitalized patients had malnutrition [26]. This trend is consistent with the increase in prevalence previously found by the largest multicenter study in Spain [15]. This finding reinforces the urgency of implementing early detection strategies and preventive nutrition policies before hospitalization, to mitigate clinical risks and improve patients’ outcomes.

Our data confirm that malnourished patients have a higher mortality rate than patients without malnutrition, and except in the case of esophageal cancer, regarding to the subgroup of patients with cancer, more malnourished patients with standard care die than those with nutritional intervention. This result confirms that malnutrition status is strongly associated with higher **mortality** [27]; this suggests that nutritional therapy should not be viewed as a supportive measure, but as an essential therapeutic intervention, and that nutrition should be integrated into routine clinical care as a proactive and essential strategy to reduce mortality.

Regarding readmission rates, the lack of impact of nutritional intervention on **90-day readmission rates** suggests limitations in the modalities used—primarily dietary counseling and oral supplements. These approaches may be inadequate for patients with complex needs, especially given their short duration and limited follow-up. More intensive, multidisciplinary strategies should be explored to improve long-term outcomes.

Regarding the comparison between patients who responded at one year, those who did not respond, and those who died, older patients showed higher mortality rates, while younger patients were more often non-responders. Those who died had lower BMI, worse HRQoL, more comorbidities, and were taking more medications at admission, highlighting that early indicators of frailty and complexity can help identify high-risk individuals and inform targeted care strategies. We observed that HRQoL (especially mental health, social functioning, physical role and vitality) was affected in patients with malnutrition at admission. Analogously, recent studies have confirmed that malnutrition has a negative physical and emotional impact on patients and impairs quality of life [28]. Malnourished patients with poor **HRQoL at admission** were the same individuals who showed a significant improvement in mental, social and physical components of quality of life at the change. Our findings are consistent with previous studies, which confirmed an increase in physical and mental scores in a nutritional intervention group of oncological [14, 29] and older patients [30, 31]. One possible explanation may be that at time of admission, their quality of life was greatly impaired; after a year, their malnutrition was more like to have improved, and consequently, their quality of life also. Therefore, it is important to acknowledge that the observed improvements in HRQoL among malnourished patients may be partially attributable to regression to the mean. Given their lower baseline scores, these individuals were statistically more likely to exhibit upward changes over time, independent of any intervention effect. While the nutritional intervention may have contributed to the positive outcomes, the influence of this statistical phenomenon cannot be excluded. Future studies incorporating control groups and extended follow-up periods would be essential to separate true intervention effects from regression artifacts. Furthermore, it is important to note that the psychological burden and social isolation associated with the COVID-19 pandemic likely influenced patient’s perception of HRQoL, independently of their clinical status, which could have amplified the observed outcomes.

Our study also identified **potential factors related to change in HRQoL** one year after admission. In terms of sociodemographic variables, some studies, similar to this one, show a decline in quality of life indicators with **aging** amongst both healthy and unhealthy elderly people [32, 33]. Another factor that might have influenced the change during the follow-up period was the fact of being **female**; this finding is consistent with those of several studies of HRQoL among elderly patients. The possible explanation is that women are more likely to report health problems [34]. Several factors may explain why women had lower HRQoL. Biologically, women are more susceptible to micronutrient deficiencies due to hormonal fluctuations, reproductive demands, and lower muscle mass, which can exacerbate fatigue, pain, and physical limitations [35]. Psychosocially, women often face greater caregiving burdens, socioeconomic disadvantages, and higher rates of anxiety and depression, all of which negatively impacts the perceived well-being [36, 37]. Furthermore, gender bias in self-reporting and societal expectations may influence how women perceive and communicate their health status [38].

In relation to the **number of medications**, Helvik et al. [39] found similar results to ours, mainly that lower physical function was significantly associated with a larger number of medications. The link between lower physical function and more medications may reflect clinical complexity and frailty. Polypharmacy can both signal and worsen physical decline, highlighting the need for integrated care and regular medication review. In our study, being hospitalized for **surgical intervention** was found to be related to worse mental health. This shows the importance of introducing regular mental health screening during the postoperative period. There are reports in the literature of attempts to introduce screening of this kind in medical oncology practice [40] but not in surgical oncology practice. Many cancer patients who undergo surgery receive their primary cancer care from their surgeon and never actually see a medical oncologist [41]. As a result, systems for screening for mental health issues in medical oncology practices may never cover patients who have had cancer operations. In future research, it would be of interest to examine mental health screening programs in surgical oncology practices. In relation to the evaluation of the **nutritional intervention** provided during the hospital stay, we found that it was not associated with better quality of life at one year, except for role physical and body pain. The review by Feinberg et al. [42] suggests that the evidence for the effects of nutritional support on quality of life is insufficient to confirm or reject clinically relevant effects of intervention on quality of life. Any nutritional intervention would probably have to be longer-lasting or even take place prior to admission. As Söderström et al. [43] state, one possible reason for the lack of significant effect observed in HRQoL among malnourished older patients receiving nutritional intervention is that it may be overconfident to expect improvements in quality of life from a nutritional intervention lasting only two weeks. This is consistent with the findings of a Cochrane review [44] which showed that the intervention time of most studies was too short to have a realistic possibility of detecting beneficial effects. Furthermore, the lack of standardized follow-up after discharge (other than advice or supplements) may explain the limited effect of the nutritional intervention. Previous research has shown that long-term, structured nutritional interventions, including multidisciplinary follow-up can significantly improve both patient nutritional status and health-related quality outcomes [45, 46]. However, it is important to note the fact the improvement of role-physical and bodily pain in malnourished patients who received nutritional intervention. For instance, Calegari et al. (2011) found that hemodialysis patients at nutritional risk who received a tailored dietary supplement improvements in subjective global assessment, physical role, and bodily pain domains of the SF-36 quality of life questionnaire in malnourished patients that received oral nutritional supplements [47]. Similarly, Gao et al. (2021) reported significant improvements in those domains in malnourished patients with intestinal failure who received home enteral nutrition [48]. These findings suggest that nutritional rehabilitation not only restores metabolic and physiological balance but also contributes meaningfully to patients’ functional capacity and pain management.

Our study has several **strengths**: (1) use of validated instruments; (2) a large sample of hospitalized patients; (3) patients with different digestive and oncological diagnoses from three different hospitals; (4) correlation of HRQoL with socio-demographic and clinical variables.

Some **limitations** should also be noted. One is the loss of participants over the course of the study. This is partly reflected in the high non-response rate at follow-up (23.78%); at the same time, any quality of life study faces the problem that the effects can only be measured among individuals who are still alive on follow-up. This limitation is particularly significant in studies such as this one, dealing with patients suffering from malnutrition, who have a high rate of mortality. Notably, patients for whom no data are available at follow-up (either because they had died or because they did not respond) were those who had the worst baseline HRQoL. It is also important to bear in mind that this study was performed during the COVID-19 pandemic, in far-from-usual clinical conditions and with many hospital restrictions on proper contact with patients. Moreover, the follow-up HRQoL measurement was performed one year after the intervention. Our results may have been conditioned by this relatively long period between baseline and follow-up measurements among patients with severe diseases who might suffer a range of adverse events during that time. The implication of non-response bias is that patients who did not complete the study could have different characteristics than those who did, affecting internal validity. Nevertheless, the analysis comparing the total patient population to the analyzed cohort (eTable 1) helps to understand potential biases. Besides, the loss of longitudinal data compromises the analysis of temporal evolution, making it difficult to identify consistent patterns. Another limitation is that, in some patients, the length of stay was very short in the intervention center, limiting the intervention period in many cases, which may not have been sufficient to cause a clinically detectable effect. On the other hand, carry out the intervention, we chose not to follow a clinical trial design; instead, we implemented the nutritional intervention at a single center, aiming for a more controlled setting while maintaining real-life clinical conditions. Finally, our intervention was performed immediately upon hospital admission, as soon as the patient was able to receive nutritional intervention This meant that among surgical patients, who are placed on a diet during the first days of admission, given that the median length of stay was 6 days, in many cases the number of days the patients could actually receive nutritional support was extremely small.

## Conclusions

While we fully agree with those authors who have stated that the real key lies in preventing malnutrition [14, 43, 44], we believe that identifying factors that influence changes in HRQoL amongst malnourished and non-malnourished patients one year after discharge might help to determine the importance of nutritional intervention in hospitalized patients. In the future, among patients with chronic pathologies or those liable to receive surgical intervention, it may be more useful to begin a nutritional intervention first by detecting possible malnutrition, long before a possible hospital admission, or as a regular treatment in these patients, and to begin this intervention immediately at home, continue it during admission and, very importantly, maintain it after discharge.

### Sources of support

This work was supported in part by grants from the Instituto de Salud Carlos III and the European Regional Development Fund (PI18/00698) “Evaluación de la efectividad y coste-efectividad de una intervención en pacientes hospitalizados con desnutrición relacionada con la enfermedad” awarded to principal investigator José Mª Quintana; the Basque Government’s Department of Health (2019111043), the Biosistemak Institute for Health Systems Research and Biocruces Bizkaia Health Research Institute; and the Instituto de Salud Carlos III’s thematic networks REDISSEC (Health Services Research on Chronic Patients Network, RD16/001/001 and RD16/0001/0009) and the Network for Research on Chronicity, Primary Care, and Health Promotion (RICAPPS, RD21/0016/0017, RD21/0016/0011, RD24/0005/0020 and RD24/0005/0019).

Note. ^1^ : Patients who responded at baseline, but not one year after admission; ^2^ : Patients who responded at the baseline and at one year after admission; ^3^ : Patients who died within one year of discharge and, therefore, responded at baseline, but not one year after admission. *Mean ± standard deviation. ^¥^Median (IQR (interquartile range)). ^†^Kruskal-Wallis test. GUH: Galdakao-Usansolo University Hospital; BUH: Basurto University Hospital; DUH: Donostia University Hospital. BMI: Body Mass Index.* P*-values in bold represent statistically significant differences at *p* = 0.05.

## Supplementary Information

Below is the link to the electronic supplementary material.

Supplementary Material 1

Supplementary Material 2

Supplementary Material 3

Supplementary Material 4

## Data Availability

The datasets generated during and/or analysed during the current study are available from the corresponding author on reasonable request.

## Abbreviations

HRQoL: Patient health-related quality of life
CEIC: Clinical research ethics committee
MUST: Malnutrition universal screening tool
GLIM: Global leadership initiative on malnutrition
VAS: Visual analogue scale
BMI: Body mass index
