# Supplementary material for: One-year post-discharge health-related quality of life in digestive and oncology patients: a three-group comparison by nutritional status and care
Source: Qual Life Res. 2025 Dec 26;35(1):10. doi: 10.1007/s11136-025-04139-y (PMC12743072; doi:10.1007/s11136-025-04139-y)
Supplement: Supplementary file 1 — Supplementary Material 1 [file 11136_2025_4139_MOESM1_ESM.docx]

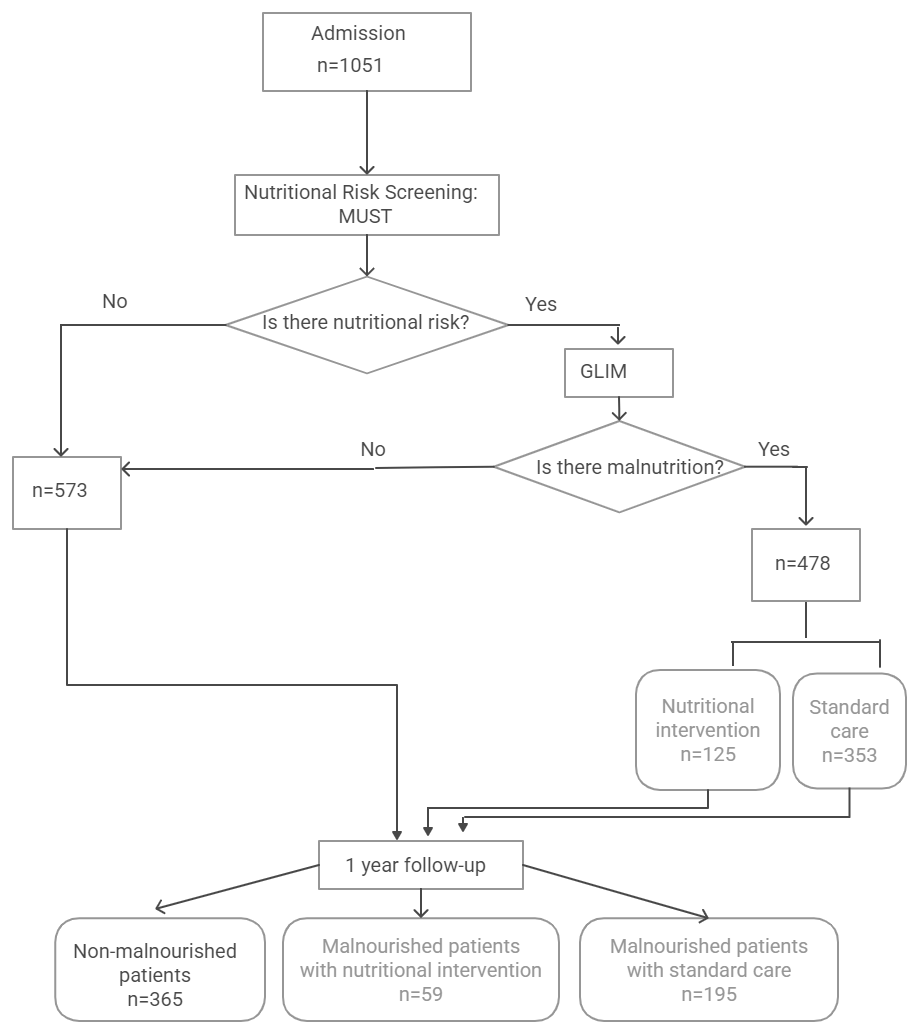


**eFigure 1. Flowchart of the procedure.**

Six hundred and nineteen patients completed the HRQoL questionnaires one year after the admission, of whom three hundred and sixty-five were non-malnourished patients, fifty nine were malnourished patients receiving nutritional intervention, and one hundred ninety-five were malnourished patients receiving standard care. HRQoL: Health-related quality of life.
