# Supplementary material for: One-year post-discharge health-related quality of life in digestive and oncology patients: a three-group comparison by nutritional status and care
Source: Qual Life Res. 2025 Dec 26;35(1):10. doi: 10.1007/s11136-025-04139-y (PMC12743072; doi:10.1007/s11136-025-04139-y)
Supplement: Supplementary file 2 — Supplementary Material 2 [file 11136_2025_4139_MOESM2_ESM.docx]

**eFigure 2.** **Comparison between initial and final evaluation for Barthel and EuroQol by malnutrition-intervention level and pathology.**

Hospitalization and last measurement points represent the median of the Barthel and EuroQol questionnaires for each group, according to the malnutrition level (0= not malnourished patients, 1= malnourished patients with nutritional intervention, and 2= malnourished patients without nutritional intervention) and to the pathology (colorectal cancer, other cancers, other pathologies). CCR: colorectal cancer. Other cancers: esophageal cancer, gastric cancer, pancreatic cancer. Other pathologies: Colitis, Crohn’s disease, pancreatitis.
