# Supplementary material for: One-year post-discharge health-related quality of life in digestive and oncology patients: a three-group comparison by nutritional status and care
Source: Qual Life Res. 2025 Dec 26;35(1):10. doi: 10.1007/s11136-025-04139-y (PMC12743072; doi:10.1007/s11136-025-04139-y)
Supplement: Supplementary file 3 — Supplementary Material 3 [file 11136_2025_4139_MOESM3_ESM.docx]

**eFigure 3.** **Comparison between initial and final evaluation for SF-36 by malnutrition-intervention level and pathology.**

Hospitalization and last measurement points represent the median domains of the SF-36 questionnaire for each group, according to the malnutrition level (0= not malnourished patients, 1= malnourished patients with nutritional intervention, and 2= malnourished patients without nutritional intervention) and to the pathology (colorectal cancer, other cancers, other pathologies). CCR: colorectal cancer. Other cancers: esophageal cancer, gastric cancer, pancreatic cancer. Other pathologies: Colitis, Crohn’s disease, pancreatitis. PF: physical function; SF: social function; MH: mental health; RE: role emotional; RP: physical role.
