# Supplementary material for: One-year post-discharge health-related quality of life in digestive and oncology patients: a three-group comparison by nutritional status and care
Source: Qual Life Res. 2025 Dec 26;35(1):10. doi: 10.1007/s11136-025-04139-y (PMC12743072; doi:10.1007/s11136-025-04139-y)
Supplement: Supplementary file 4 — Supplementary Material 4 [file 11136_2025_4139_MOESM4_ESM.docx]

**eTable 1. Comparison of the type of admitted patient according to response**.

| **Variables** | **Total (n=1051)** | **Non responders^1^ (n=250)** | **Responders^2^ (n=619)** | **Deceased^3^ (n=182)** | **P value** |
| --- | --- | --- | --- | --- | --- |
| *Sociodemographic and clinical data* |  |  |  |  |  |
| Age* | 62.7±16.3 | 56.2±16.9^2.3^ | 63.5±16.3^1.3^ | 69.0±12.2^1.2^ | **<.0001**^†^ |
| Gender (Male) | 640 (60.9) | 138 (55.2) | 381 (61.6) | 121 (66.5) | 0.05 |
| Main pathology |  | ^2,3^ | ^1,3^ | ^1,2^ |  |
| Esophageal cancer | 30 (2.9) | 3 (1.2) | 12 (1.9) | 15 (8.2) | 0.42 |
| Not malnourished | 7 (23.3) | 1 (33.3) | 3 (25.0) | 3 (20.0) |  |
| Malnourished with nutritional intervention | 8 (26.7) | 1 (33.3) | 1 (8.3) | 6 (40.0) |  |
| Malnourished with standard care | 15 (50.0) | 1 (33.3) | 8 (66.7) | 6 (40.0) |  |
| Gastric cancer | 113 (10.8) | 17 (6.8)^3^ | 56 (9.1)^3^ | 40 (22.0)^1,2^ | **0.02** |
| Not malnourished | 43 (38.1) | 10 (58.8) | 26 (46.4) | 7 (17. 5) |  |
| Malnourished with nutritional intervention | 22 (19.5) | 2 (11.8) | 10 (17.9) | 10 (25.0) |  |
| Malnourished with standard care | 48 (42.5) | 5 (29.4) | 20 (35.7) | 23 (57.5) |  |
| Pancreatic cancer | 90 (8.6) | 13 (5.2) | 27 (4.4) | 50 (27.5) | 0.99 |
| Not malnourished | 28 (31.1) | 4 (30.8) | 9 (33.3) | 15 (30.0) |  |
| Malnourished with nutritional intervention | 24 (26.7) | 4 (30.8) | 7 (25.9) | 13 (26.0) |  |
| Malnourished with standard care | 38 (42.2) | 5 (38.5) | 11 (40.7) | 22 (44.0) |  |
| Colorectal cancer | 381 (36.3) | 80 (32.0)^3^ | 229 (37.0) | 72 (39.6)^1^ | **0.02** |
| Not malnourished | 205 (53.8) | 50 (62.5) | 126 (55.0) | 29 (40.3) |  |
| Malnourished with nutritional intervention | 41 (10.8) | 11 (13.8) | 22 (9.6) | 8 (11.1) |  |
| Malnourished with standard care | 135 (35.4) | 19 (23.8) | 81 (35.3) | 35 (48.6) |  |
| Colitis | 75 (7.1) | 22 (8.8) | 53 (8.6) | 0 (0.0) | 0.34 |
| Not malnourished | 32 (42.7) | 8 (36.4) | 24 (45.3) | 0 (0.0) |  |
| Malnourished with nutritional intervention | 13 (17.3) | 6 (27.3) | 7 (13.2) | 0 (0.0) |  |
| Malnourished with standard care | 30 (40.0) | 8 (36.4) | 22 (41.5) | 0 (0.0) |  |
| Crohn's | 99 (9.4) | 29 (11.6) | 69 (11.2) | 1 (0.6) | 0.39 |
| Not malnourished | 53 (53.5) | 15 (51.7) | 38 (55.1) | 0 (0.0) |  |
| Malnourished with nutritional intervention | 5 (5.1) | 0 (0.0) | 5 (7.3) | 0 (0.0) |  |
| Malnourished with standard care | 41 (41.4) | 14 (48.3) | 26 (37.7) | 1 (100.0) |  |
| Pancreatitis | 263 (25.0) | 86 (34.4) | 173 (28.0) | 4 (2.2) | 0.73 |
| Not malnourished | 205 (78.0) | 63 (73.3) | 139 (80.4) | 3 (75.0) |  |
| Malnourished with nutritional intervention | 12 (4.6) | 5 (5.8) | 7 (4.1) | 0 (0.0) |  |
| Malnourished with standard care | 46 (17.5) | 18 (20.9) | 27 (15.6) | 1 (25.0) |  |
| Surgery intervention | 479 (45.6) | 140 (56.0)^3^ | 305 (49.3)^3^ | 34 (18.7)^1,2^ | **<.0001** |
| Charlson Comorbidity Index^¥^ | 2.0 (0.0–3.0) | 2.0 (0.0–3.0)^3^ | 2.0 (0.0–3.0)^3^ | 6.0 (2.0–7.0)^1,2^ | **<.0001^†^** |
| Number of drugs^¥^ | 4.0 (2.0–7.0) | 3.0 (1.0–6.0)^3^ | 4.0 (2.0–7.0)^3^ | 6.0 (3.0–9.0)^1,2^ | **<.0001^†^** |
| *Nutritional variables* |  |  |  |  |  |
| GLIM |  |  |  |  | **< 0.0001** |
| Not malnourished | 573 (54.53) | 151 (60.40) | 365 (28.97) | 57 (31.32) |  |
| Malnourished with nutritional intervention | 125 (11.89 | 29 (11.60) | 59 (9.53) | 37 (20.33) |  |
| Malnourished with standard care | 353 (33.59) | 70 (28.00) | 195 (31.50) | 88 (48.35) |  |
| Handgrip strength at admission* | 27.1±11.7 | 28.6±12.6^3^ | 27.4±11.6^3^ | 24.0±10.2^1.2^ | **0.0004^†^** |
| Calf at admission* | 35.4±3.8 | 35.9±3.7^3^ | 35.6±3.7^3^ | 33.9±3.9^1.2^ | **<.0001^†^** |
| Weight at admission* | 71.2±15.9 | 72.9±16.2^3^ | 71.9±16.0^3^ | 66.7±14.2^1.2^ | **<.0001^†^** |
| BMI at admission* | 25.6±5.1 | 26.0±5.1^3^ | 25.8±5.2^3^ | 24.2±4.7^1.2^ | **0.0001^†^** |
| Days of admission^¥^ | 6.0 (4.0–8.0) | 5.5 (4.0–9.0) | 6.0 (4.0–8.0) | 6.0 (3.0–9.0) | 0.72^†^ |
| Infectious complications | 37 (3.5) | 8 (3.2) | 18 (2.9) | 11 (6.0) | 0.12 |
| *Health-related Quality of life questionnaires at baseline* | | | | | |
| Barthel* | 94.5±10.6 | 95.1±9.1^3^ | 95.5±8.7^3^ | 89.7±16.1^1.2^ | **<.0001^†^** |
| EQ-5D* | 0.8±0.2 | 0.8±0.2^3^ | 0.8±0.2^3^ | 0.7±0.2^1.2^ | **<.0001^†^** |
| SF-36 Mental Health* | 60.1±18.1 | 58.1±17.9^2^ | 62.2±17.8^1,3^ | 55.8±18.7^2^ | **<.0001^†^** |
| SF-36 Physical Functioning* | 66.5±30.3 | 70.1±30.0^3^ | 70.4±28.2^3^ | 48.4±31.0^1.2^ | **<.0001^†^** |
| SF-36 Social Functioning* | 63.4±33.0 | 61.8±32.8^2.3^ | 68.2±31.0^1.3^ | 49.3±35.7^1.2^ | **<.0001^†^** |
| SF-36 Physical Role* | 57.5±35.1 | 56.3±34.4^2.3^ | 63.1±33.8^1.3^ | 40.3±34.9^1.2^ | **<.0001^†^** |
| SF-36 Emotional Role* | 79.3±27.2 | 76.0±27.5^2^ | 82.6±24.5^1,3^ | 72.0±33.3^2^ | **0.0002^†^** |
| SF-36 Vitality* | 49.3±21.1 | 47.8±19.8^2.3^ | 52.9±20.6^1.3^ | 39.5±21.0^1.2^ | **<.0001^†^** |
| SF-36 Body Pain* | 54.7±34.2 | 52.4±34.4^2.3^ | 58.8±33.3^1.3^ | 43.8±34.2^1.2^ | **<.0001^†^** |
| SF-36 General Health* | 49.5±20.3 | 48.6±20.2^2.3^ | 53.1±19.6^1.3^ | 38.8±18.7^1.2^ | **<.0001^†^** |

  Note. ^1^ : Patients who responded at baseline, but not one year after admission; ^2^ : Patients who responded at the baseline and at one year after admission; ^3^  : Patients who died within one year of discharge and, therefore, responded at baseline, but not one year after admission. *Mean ± standard deviation. ^¥^Median (IQR (interquartile range)). ^†^Kruskal-Wallis test. GUH: Galdakao-Usansolo University Hospital; BUH: Basurto University Hospital; DUH: Donostia University Hospital. BMI: Body Mass Index. P-values in bold represent statistically significant differences at p=0.05
